# The Seoul Metropolitan Lifestyle Intervention Program and Metabolic Syndrome Risk: A Retrospective Database Study

**DOI:** 10.3390/ijerph13070667

**Published:** 2016-07-04

**Authors:** Jina Choo, Seok-Jun Yoon, Hosihn Ryu, Mi-Suk Park, Hyang Sook Lee, Yoo Mi Park, Do-Sun Lim

**Affiliations:** 1 College of Nursing, Korea University, Seoul 02841, Korea; hosihn@korea.ac.kr; 2 Department of Preventive Medicine, Korea University College of Medicine, Seoul 02841, Korea; yoonsj02@korea.ac.kr; 3 Metabolic Syndrome Management Center of Seoul Metropolitan Government, Seoul 02751, Korea; 386now00@hanmail.net; 4 Medical and Health Policy Division, Seoul Metropolitan Government, Seoul 04524, Korea; lhs100@seoul.go.kr (H.S.L.); yoom66@seoul.go.kr (Y.M.P.); 5 Department of Cardiology, School of Medicine, Korea University, Seoul 02841, Korea; dslmd@kumc.or.kr

**Keywords:** metabolic syndrome, lifestyle, cardiovascular diseases, community health service, prevention

## Abstract

Since 2011, the Seoul Metabolic Syndrome Management (SMESY) program has been employed as a community-wide, lifestyle modification intervention in Seoul, Korea. We aimed to determine if the SMESY intervention would be significantly associated with improvements in metabolic syndrome (MetS) risk factors. This retrospective database study included data from 25,449 participants aged 30–64 years between 1 January 2013 and 30 June 2013. In the SMESY program, 3 risk-stratified groups by the number of MetS factors were followed for 12 months with different intensity and timeframe of intervention. Among the high-(*n* = 7116) and moderate-risk groups (*n* = 14,762), all MetS factors (except triglycerides among the moderate-risk group) as well as MetS z-scores significantly improved over 12 months (all *p* < 0.05). Among the low-risk group (*n* = 3571), all factors aggravated significantly over 12 months (all *p* < 0.05). We observed temporal associations between the implementation of the SMESY program and improvements in MetS risk factors. However, such improvements differed by risk-stratified group, being most robust for the high-risk group, modest for the moderate-risk group, and aggravated for the low-risk group. Thus, more intensive interventions targeting different risk-stratified groups are needed, given a better understanding of the increase in risk factors observed in the low-risk group.

## 1. Introduction

Metabolic syndrome is a public health concern in countries across all economic strata. The overall prevalence of metabolic syndrome among adults has increased from 32.9% to 36.1% in the United States [1], and from 29.2% to 31.3% in Korea [2] during the period 2001–2008. Metabolic syndrome is associated with an increased risk of cardiovascular disease (CVD), which is the global leading causes of death with a high economic burden [3,4]. Increasing medical expenditure on CVD has resulted in large social and economic burdens for most countries, including Korea. This is partly due to increases in populations vulnerable to unhealthy behavioral/lifestyle factors [5] and increasing elderly populations [6]. Hence, identification and management of metabolic syndrome may play a critical role in reducing the risk of and medical expenditure on CVD at population level.

Population-based strategies for CVD prevention have been consistently urged [7]. The World Health Organization (WHO) recommends population-based CVD prevention strategies by utilizing the concept of metabolic syndrome, from the standpoint that metabolic syndrome is a pre-morbid state of CVD and diabetes rather than a clinical diagnosis [8]. In this context, a community-wide program on the identification and management of metabolic syndrome—The Seoul Metabolic Syndrome Management [SMESY] program—was officially implemented in 2011. In this community-wide model, standardized lifestyle interventions were instituted at public health centers across 25 municipal districts in Seoul [9]. The SMESY program is a free healthcare service for Seoul citizens aged 30–64 years, funded and supervised by the Seoul Metropolitan Government. Before launching the program, the prevalence of metabolic syndrome in the Korean population group aged 20–29 years (10.9%) was approximately half that of the population group aged 30–39 years (21.6%) [10]. Targeting the general population aged 30–64 years, the SMESY program focuses on risk-stratified, nurse-coordinated, comprehensive lifestyle modifications including diet, physical activity, and weight loss interventions with periodic follow-ups over 12 months. The rationale and preliminary results of the SMESY program were reported using the 2011–2012 data after the project was initially launched [9].

Lifestyle modifications may form an integral part of cardiovascular risk reduction programs. Two systematic reviews have reported that lifestyle interventions, such as either combined diet-plus-exercise interventions or diet-only interventions, had beneficial effects on the reduction of some risk factors of metabolic syndrome, including waist circumference (WC) and triglyceride levels, when conducted for either 6 or 12 months [11,12]. Moreover, a 12-month comprehensive lifestyle intervention covering diet, exercise, and behavioral modifications, significantly reduced the prevalence of metabolic syndrome with 31% absolute risk reduction [13]. However, given the current evidence concerning the efficacy of lifestyle interventions on the risk for metabolic syndrome, whether a community-wide lifestyle intervention would have significant benefits for risk reduction of metabolic syndrome needs to be evaluated in real community settings, rather than in research-based, controlled settings.

Therefore, the present study aimed to determine if a 12-month SMESY lifestyle intervention was significantly associated with improvements in risk factors of metabolic syndrome (WC, high blood pressure, dyslipidemia, and glucose intolerance) and in behavioral lifestyle factors (smoking, physical activity, healthy diet, and body weight).

## 2. Materials and Methods

### 2.1. Study Design and Setting

This is a retrospective database study, which included the 2013 data of the SMESY program [9]. The study had a longitudinal pre- and post-test design to investigate time effects of the SMESY intervention on metabolic risk. It was retrospectively conducted with a cohort recruited from 25 public health centers in Seoul.

Seoul is the capital and largest metropolis of Korea. It has a population of 10,369,000 people and covers a metropolitan area of 605 km^2^ [2,14]. Seoul is divided into 25 municipal districts (called “Gu” in Korean), which range in size from 136,000 to 672,000 people. Each municipal district has a public health center that is responsible for providing primary health care to district residents.

### 2.2. Study Participants

Participants registered in the SMESY program during the year 2013 were 176,321 citizens; the eligibility criteria of the participant of the SMESY program were to be men and women residents of each municipal district, voluntarily attending the program, and being 30–64 years old. Of those registered during the year 2013 (*N* = 176,321), we recruited 89,997 who were registered from 1 January 2013 to 30 June 2013. Of these 89,997 participants, we excluded those who violated the frequencies of designated follow-up (*n* = 48), had clinical diseases such as cardiovascular disease, cancer, or kidney diseases (*n* = 1610), followed up less than twice (*n* = 50,983), and had coding errors (*n* = 4188). In the SMESY program, participants are divided into four risk-stratified groups [9]. We excluded one of these risk-groups: the disease group (i.e., the Motivational-B group [9]). This group comprises those with either hypertension or diabetes, or those taking any medication for hypertension, diabetes, or dyslipidemia (*n* = 7719). After all exclusions, 25,449 participants were included in the present study. These participants were categorized into three risk-stratified groups: high-risk (*n* = 7116), moderate-risk (*n* = 14,762), and low-risk (*n* = 3571), determined by the number of metabolic syndrome risk factors present. In the SMESY program, metabolic syndrome and its risk factors are defined using the revised criteria of the third report of the National Cholesterol Education Program (NCEP) Expert Panel on Detection, Evaluation, and Treatment of High Blood Cholesterol in Adults (Adult Treatment Panel [ATP] III) [15]: Abdominal obesity (determined by increased WC), raised triglyceride levels, reduced high-density lipoprotein cholesterol (HDL-C) levels, high blood pressure, and high fasting glucose levels. Increased WC was defined as >90 cm for men and >85 cm for women, according to the criteria defined by the Korean Society for the Study of Obesity [16]. The high-risk group was defined as those with ≥3 risk factors (i.e., those with metabolic syndrome). The moderate-risk group was defined as those with 1 or 2 risk factors. The low-risk group included participants with no risk factors.

All participants were explained that their clinical data might be used for academic purposes, and informed consent was taken from each participant. Participants were asked to complete routine questionnaire packages, including sociodemographic and health-related lifestyle variables. The study was approved by the Institutional Review Board at Korea University (KU-IRB-15-EX-253-A-1).

### 2.3. SMESY Intervention

The SMESY intervention focuses on the screening and management of metabolic syndrome by trained health professionals (i.e., nurse coordinators, dieticians, exercise specialists, and doctors if needed). With regard to the screening, five risk factors of metabolic syndrome were measured after participants completed their questionnaire packages. Nurse coordinators reviewed the clinical results, and determined which risk-stratified group the participants would be allocated to, based on the standardized manual of the SMESY program [9]. The management of metabolic syndrome was achieved through different intensity and timeframe of intervention by risk-stratified groups; detailed information is described elsewhere [9]. Intervention components of the SMESY program include face-to-face counseling on healthy diet and exercise, and cell-phone text messaging about behavioral modifications. All risk-stratified groups are planned to be followed up for up to 12 months. The specific follow-up timeframes were 0, 3, 6, 9, and 12 months for the high-risk group (face-to-face counseling every 3 months and weekly text messaging); 0, 6, and 12 months for the moderate-risk group (face-to-face counseling every 6 months and text messaging once every two weeks); and 0 and 12 months for the low-risk group (face-to-face counseling at baseline and monthly text messaging). For example, a citizen with metabolic syndrome undergoes screening for risk factors of metabolic syndrome and receives initial face-to-face counseling. This counseling includes comprehensive risk reduction provided by nurse coordinators, dietary modification by dieticians, and exercise coaching by exercise specialists. A further four follow-up visits are recommended over the next 12 months. At the month 3, 6, 9, and 12 visit, the participant undergoes screening and receives further face-to-face counseling. Using an inventory of text messages developed according to the risk-stratification category, text messaging is set up by nurse coordinators and sent via cell phones over 12 months. The text-messages include advice on healthy diet, regular exercise, smoking cessation, moderation in drinking alcohol, and stress management techniques.

### 2.4. Outcome Measures

In the present study, the outcome measures were: (1) the levels of risk factors of metabolic syndrome as used by continuous variables (i.e., WC, systolic and diastolic blood pressures [BP], HDL-C, triglycerides, and fasting glucose); (2) integrated risk scores of metabolic syndrome as measured by z-scores (i.e., risk scores of metabolic syndrome); (3) behavioral lifestyle factors such as current smoking (%), physical activity (%), healthy diet behaviors (scores), and body weight (kg); and (4) the prevalence of metabolic syndrome (%). Risk scores for metabolic syndrome were obtained as the sum of standardized scores of WC, mean arterial pressure, HDL-C, triglycerides, and fasting glucose, calculated by subtracting the individual risk factor from the NCEP ATP III criteria and dividing that by the sample standard deviation [17]. Mean arterial pressure was obtained from the values of systolic and diastolic BPs based on the formula:

mean BP = (1/3) × (systolic BP − diastolic BP) + diastolic BP(1)

The prevalence of metabolic syndrome at each time point was obtained as the percentage of participants with metabolic syndrome of those who completed follow-up at each time point. Physical activity was defined as adequate levels of physical activity, assessed as participating in moderate-intensity exercise for ≥30 min per session on ≥5 days per week (yes = 1, no = 0). Healthy diet behaviors (i.e., healthy diet scores) were assessed by a 10 item-scale with two response options; 0 = to fulfill on less than 5 days per week vs. 1 = to fulfill on five or greater days per week (e.g., I have a serving of colorful vegetables two times per day). The total score range is 0–10.

All blood samples for measures of metabolic risk factors were obtained at 25 public health centers in the morning after a 10-h overnight fast, prior to taking current medication(s). Either venipuncture (two centers) or point of care testing (23 centers) were used for the measurements of triglycerides and HDL-C. The point of care testing systems included LipidPro [18], LABGEO PT10 [19], and LDX^®^ system [20] which had <7% of inter-assay and intra-assay coefficients of variation. Body weight (kg) was measured after an overnight fast, using the bioelectrical impedance scale. Height (cm) was measured using a wall-mounted stadiometer. Body mass index (BMI) was computed as weight (kg)/height (m)^2^. WC (cm) was measured twice using a measuring tape at the midpoint between the lowest rib and the iliac crest; the average of two measurements was used. Blood pressure was measured on the right arm in the sitting position after a 10-min rest with the bladder emptied, by using an automated sphygmomanometer.

### 2.5. Data Analysis

All data were analyzed using SPSS 23.0 (SPSS Inc., Chicago, IL, USA). A *p*-value < 0.05 was considered statistically significant. Participants’ general characteristics were expressed as either means (standard deviations, SD) or numbers (%) for total participants as well as for risk-stratified groups. Group differences in all participants’ sociodemographic and health-related characteristics were analyzed with the analysis of variance (ANOVA) or Chi-square test, as appropriate.

To examine the main effects of time on the repeated measures of the risk factors and risk scores of metabolic syndrome, behavioral lifestyle factors (i.e., healthy diet scores and body weight) were periodically followed up for 12 months; the linear mixed model with fixed effects for intercepts and time was performed. Additionally, to account for the non-linear trend in time, the fixed effects for time were included using categorical variables of time. Moreover, in order to examine the main effects of time on behavioral lifestyle factors (i.e., current smoking and physical activity) over the periodic follow-up of 12 months and on changes in the prevalence of metabolic syndrome for a pooled group (i.e., the combination of three risk-stratified groups) from baseline time point to 12-month time point, the generalized estimating equation (GEE) was performed. The linear mixed and GEE models used in these analyses were all adjusted for age, sex, education, income, marital status, current smoking status, physical activity, alcohol drinking, BMI at baseline, and medication status (i.e., taking medications for hypertension, diabetes, or dyslipidemia) over 12 months.

## 3. Results

### 3.1. Participants’ Characteristics

Participants (*N* = 25,449) had a mean age of 50.0 years, with a higher proportion of women (62.6%) (Table 1). Overall, 83.6% of participants had college education or higher, 20.1% had a monthly household income of <2,000,000 won (approximately $1818), and 2.9% had medical aid cover (i.e., a public medical assistance program targeted at those of low socioeconomic status). The high- and moderate-risk groups had a greater mean age than the low-risk group (*p* < 0.001) and were more likely to be socioeconomically vulnerable, specifically having a greater proportion of participants with a monthly household income of <2,000,000 won and with medical aid cover (*p* < 0.001).

The cut-off value of 2,000,000 won was based on the minimum cost of living, as defined by the Ministry of Health and Welfare in Korea (1,630,820 won) [21]. Korean currency indicates that a million won is approximately equal to 900 US dollars [22]. Of the total participants, 11.1% were current smokers, 9.5% reported doing physical activity, and 29.8% reported drinking alcohol ≥ 2 times per week (Table 1). The participants had a mean BMI of 24.1 kg/m^2^ and a mean diet score of 6.8 points (range 0–10 points). 

### 3.2. Changes in the Risk Factors of Metabolic Syndrome: Time Effects by Group

Among the high-risk group, all the risk factors of metabolic syndrome significantly improved over the 12 months (Table 2) (all *p* < 0.001).

Among the moderate-risk group, WC, systolic and diastolic BPs, and fasting glucose levels significantly decreased and HDL-C significantly increased over the 12 months (all *p* < 0.05); however, triglyceride levels did not show any significant changes over 12 months. Meanwhile, among the low-risk groups, all the risk factors of metabolic syndrome significantly aggravated over the 12 months (all *p* < 0.001).

### 3.3. Changes in the Risk Scores of Metabolic Syndrome: Time Effects by Group

Finally, the risk scores of metabolic syndrome, as measured by z-scores, decreased significantly among the high- and moderate-risk groups (all *p* < 0.001), but increased significantly among the low-risk group over 12 months (*p* < 0.001) (Table 3). Among the high-risk group, the risk scores for metabolic syndrome decreased most at 3 months and showed a gradual increasing trend over the subsequent 6 months. Among the moderate-risk group, these scores decreased most at 6 months, and appeared to increase over the next 6 months.

### 3.4. Changes in Behavioral Lifestyle Factors 

The proportion of current smokers decreased significantly over 12 months among the high- and moderate-risk groups (all *p* < 0.001), but not among the low-risk group (Table 4). Among each group, the proportion of participants who engaged in adequate levels of physical activity increased significantly (all *p* < 0.001), and healthy diet scores increased significantly over 12 months (all *p* < 0.001). Among the high-risk group, the healthy diet scores increased significantly from 6.42 points to 7.58 points between baseline and 12 months. Finally, body weight (kg) decreased significantly among the high- and moderate-risk groups (all *p* < 0.001), but increased significantly among the low-risk group over 12 months (*p* < 0.001). 

### 3.5. Changes in the Prevalence of Metabolic Syndrome

**Figure 1:**
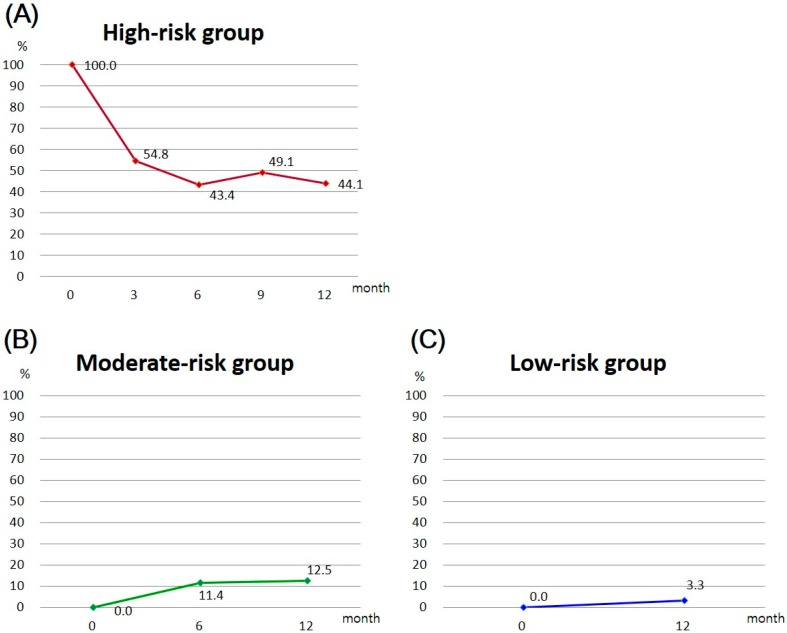
Changes in the prevalence of metabolic syndrome by group, over the SMESY intervention: (**A**) Changes in the prevalence of metabolic syndrome in the high-risk group; (**B**) Changes in the prevalence of metabolic syndrome in the moderate-risk group; (**C**) Changes in the prevalence of metabolic syndrome in the low-risk group.

Figure 1 shows the changes in the prevalence of metabolic syndrome by group over the 12-month SMESY intervention. Among the high-risk group, the prevalence of metabolic syndrome decreased gradually over 12 months, that is, from 100% to 44.1% (Figure 1A). However, the prevalence increased among the moderate- and low-risk groups by 12.5% and by 3.3%, respectively (Figure 1B,C). When the three risk-stratified groups were pooled into a group, the prevalence decreased significantly from 28.0% to 10.7% between baseline and 12 months (odds ratio = 0.51, confidence interval = 0.476–0.547, *p* < 0.001) (Table 5).

## 4. Discussion

We observed temporal associations between the implementation of the SMESY program and improvements in the metabolic syndrome risk. Substantial improvements were observed for favorable changes in mean values of the risk factors and z-scores of metabolic syndrome, the prevalence of metabolic syndrome, and behavioral lifestyle factors. However, such improvements differed by risk-stratified group, being most robust for the high-risk group, modest for the moderate-risk group, and aggravated for the low-risk group.

We found significant improvements in mean values of all the risk factors for metabolic syndrome among the high-risk group (participants with metabolic syndrome), which is the main target of the SMESY program. For example, the SMESY intervention in a real-world setting led to a 2.0 cm reduction in mean waist circumference among individuals with metabolic syndrome, only slightly less than that (−2.7 cm) in a controlled setting, as reported by Yamaoka et al. in a systematic review of the effects of lifestyle modification for individuals with metabolic syndrome [11].

These improvements in the mean values of the risk factors may have an implication, according to the notion of Rose, that shifting the risk distribution in a population may have a beneficial effect on preventing morbidity and mortality [23,24], and may, moreover, result from the beneficial effects of intensive lifestyle modifications, such as favorable changes in current smoking, physical activity, healthy diet behaviors, and weight loss. The 12-month lifestyle modification in the high-risk group may have been achieved through the use of behavioral strategies such as weekly mobile phone messaging as well as face-to-face counseling at intervals of 3 months. Previously, a systematic review revealed that technology was a useful adjunct in improving patient motivation when combined with a face-to-face relationship [25].

Furthermore, we discuss two points based on the findings of the high- and moderate-risk groups. First, significant improvements in behavioral lifestyle factors showed the most prominent improvement at 3 months that slowly changed or re-bounded slightly over the next 9 months; such a time trend was comparable with that for the risk scores of metabolic syndrome over the entire period of 12 months. This trend was similarly shown in the moderate-risk group (prominent improvements in the risk scores of metabolic syndrome at 6 months with subsequent slowdown). Second, despite favorable outcomes of the SMESY interventions in the high-risk group, the control of triglycerides and fasting glucose levels was sub-optimal at the final follow-up visit, at which point they were higher than 150 mg/dL and 100 mg/dL, respectively. These two points suggest the need for more intensive interventions after the first 3 months of the program. Based on the premise of the relationship between motivation and behavior, a decrease in motivation for maintaining healthy lifestyle habits may partially explain the slowdown in improvements in behavioral lifestyle factors and metabolic risk scores observed after the first 3 months. Teixeira et al. elicited from a systematic review that intrinsic motivation was predictive of long-term exercise adherence [26]. One effective strategy to increase intrinsic motivation addressed in the literature was motivational interviewing [27]. Rubak et al. reported from a meta-analysis that motivational interviewing has beneficial effects on BMI, total cholesterol, and systolic blood pressure [28]. Thus, motivational interviewing should be adopted as an integral part of the face-to-face counseling sessions after the first 3 months of the SMESY program. Moreover, Liu et al. reported that four interactive group discussion sessions with weekly phone contact over 6 months had beneficial effects on body weight reduction and metabolic syndrome prevalence [29]. Adding interactive interventions to the overall SMESY program may help to prolong its benefits. Finally, motivational interviewing and interactive discussions may be interventional components to be considered for increasing long-term effects of the SMESY intervention; their beneficial effects on the metabolic risk should be examined in future studies.

We found significant improvements in all the factors of metabolic syndrome, except triglycerides, among the moderate-risk group. The non-significant changes in triglycerides levels could be attributed to two possibilities: one may be a ceiling effect due to relatively desirable levels of triglycerides at baseline (122.9 mg/dL). The other may be a delaying effect resulting from delayed aggravations, as observed in the low-risk group.

Notably, the low-risk group—Who received a single follow-up at 12 months after enrollment—showed significantly aggravated levels in all factors of metabolic syndrome. For example, the systolic and diastolic blood pressure measurements were significantly elevated by an average of 1.30 mmHg and 0.80 mmHg, respectively. Although these changes are small, and the values remain within desirable limits, the aggravated changes may have significant long-term clinical implications. This finding indicates that favorable impact of baseline interventions may not last up to one year, and the aggravated progress of natural diversity may be on the secular trend. Hence, implementation of stronger preventive strategies via more frequent interactive contacts and counseling is critical. In addition, future studies are needed to identify this trend.

This study has several strengths. First, to the best of our knowledge, this is the first study to report the temporal associations between a community-wide (i.e., metropolitan-wide) lifestyle intervention program and the risk for metabolic syndrome in a real-world setting. Second, the results of changes in self-reported behavioral factors (current smoking, physical activity, and healthy diet) were assessed concomitantly with risk factors of metabolic syndrome. Third, one-year patterns of time effects of risk- and behavioral lifestyle factors of metabolic syndrome were assessed by using repeated measures over a 12-month period. In this context, our findings may provide a basis for the sustainability and dissemination of the SMESY program.

Several limitations must be considered. First, the study design—A retrospective, non-experimental study without a control group—cannot guarantee a causal connection between the SMESY interventions and cardiometabolic outcomes, because of the diffusion possibility that participants might have had additional access to other interventions. However, since no other community-wide, lifestyle intervention programs comparable with the SMESY program were available contemporarily in Seoul, other interventions accessible to citizens would likely be rare. A randomized controlled trial is needed to test the effects of the SMESY program. Second, we excluded individuals who did not visit the SMESY program after the baseline visit (dropouts, 52.6% of total participants). This may have led to biased results regarding changes in metabolic syndrome risk. Therefore, community-based strategies for increasing adherence rates are needed in the SMESY program. Third, the present study used the NCEP-ATP III criteria to diagnose metabolic syndrome. Organizations, including the International Diabetes Federation (IDF), use different criteria [30]. The prevalence of metabolic syndrome assessed using the NCEP-ATP III criteria may be different from that assessed by using other organizations’ criteria [10]. In this regard, our results of the prevalence of metabolic syndrome should be carefully interpreted. Finally, 83.6% of the study participants had college education or higher, which resulted in selection bias. Lower levels of education may be a predictor of adherence to long-term therapies [31,32]. Based on this, the study participants might have had positive benefits of the reductions in metabolic risk that may be associated with adherence.

## 5. Conclusions

The community-wide SMESY program was associated with improvements in risk factors and behavioral lifestyle factors of metabolic syndrome. These findings will provide underlying data for not only optimizing the model of the SMESY program on prevention and management of metabolic syndrome, but also for sustaining and expanding the SMESY program nationwide in Korea.

## Figures and Tables

**Table 1:** General characteristics of participants, stratified by group (*N* = 25,449).

Characteristics	n (%) or Mean (SD)					*p* *
	N	Total	High-Risk Group (*n* = 7116)	Moderate-Risk Group (*n* = 14,762)	Low-Risk Group (*n* = 3571)	
**Sociodemographic characteristics:**						
Age, years	25,449	50.0 (9.40)	50.5 (9.17) ^b^	50.2 (9.32) ^c^	48.2 (9.97)	<0.001
Female, yes	25,449	15,937 (62.6)	3556 (50.0)	9595 (65.0)	2786 (78.0)	<0.001
Education, yes	23,611					
Some college or greater		19,747 (83.6)	5345 (80.6)	11,507 (84.0)	2895 (88.0)	<0.001
High school degree or lower		3864 (16.4)	1283 (19.4)	2185 (16.0)	396 (12.0)	
Monthly household income, yes	24,319					<0.001
<2,000,000 won		4886 (20.1)	1485 (21.9)	2827 (20.1)	574 (16.6)	
>2,000,000 won		19,433 (79.9)	5288 (78.1)	11,252 (79.9)	2893 (83.4)	
Marital status, yes	25,178					0.002
Married		22,097 (87.8)	6264 (88.5)	12,797 (87.8)	3036 (86.1)	
Widow/Divorced/Separated/Single		3081 (12.2)	816 (11.5)	1,776 (12.2)	486 (13.9)	
Coverage of health security, yes	25,137					<0.001
Health insurance		24,401 (97.1)	6823 (96.5)	14,125 (97.1)	3453 (98.0)	
Medical aid		736 (2.9)	247 (3.5)	417 (2.9)	72 (2.0)	
**Health-related characteristics:**						
Current smoking, yes	25,180	2872 (11.4)	1226 (17.3)	1466 (10.1)	180 (5.1)	<0.001
Physical activity, yes	25,329	2299 (9.1)	555 (7.8)	1389 (9.5)	335 (10.0)	<0.001
Alcohol drinking, yes	14,802	4257 (28.8)	1581 (36.9)	2266 (26.9)	410 (19.7)	<0.001
Healthy diet score	25,246	6.8 (2.33)	6.4 (2.36) ^a,b^	6.9 (2.30) ^c^	7.0 (2.32)	<0.001
BMI, kg/m^2^	25,449	23.8 (3.15)	25.9 (3.20) ^a,b^	23.4 (2.76) ^c^	21.7 (2.25)	<0.001

BMI: Body mass index; SD: Standard deviation; Physical activity: Moderate intensity activity ≥5 days per week; Alcohol drinking: Drinking ≥2 times per week; * Significance for between-group difference using either ANOVA or Chi-square test; ^a^ *p* < 0.05 Scheffe post hoc test for mean difference between high-risk and moderate-risk groups; ^b^ *p* < 0.05 Scheffe post hoc test for mean difference between high-risk and low-risk groups; ^c^ *p* < 0.05 Scheffe post hoc test for mean difference between moderate-risk and low-risk groups.

**Table 2:** Changes in risk factors of metabolic syndrome over the SMESY intervention (*N* = 25,449).

Time (Month)	High-Risk Group (*n* = 7116)		Moderate-Risk Group (*n* = 14,762)		Low-Risk Group (*n* = 3571)	
	Mean (SD)	*p* *	Mean (SD)	*p* *	Mean (SD)	*p* *
WC, cm		<0.001		<0.001		0.006
0M	88.9 (7.78)		81.2 (7.61)		75.6 (6.29)	
3M	87.6 (7.58) ^a^		-		-	
6M	87.2 (7.36) ^b^		80.8 (7.27) ^b^		-	
9M	87.2 (7.56) ^c^		-		-	
12M	86.9 (7.38) ^d^		80.9 (7.31) ^d^		76.2 (6.77) ^d^	
SBP, mmHg		<0.001		<0.001		<0.001
0M	132.7 (15.52)		122.3 (14.80)		112.2 (9.90)	
3M	125.9 (13.74) ^a^		-		-	
6M	126.2 (13.35) ^b^		120.1 (13.34) ^b^		-	
9M	127.2 (13.87) ^c^		-		-	
12M	126.9 (13.38) ^d^		120.7 (13.34) ^d^		113.5 (11.22) ^d^	
DBP, mmHg		<0.001		<0.001		<0.001
0M	84.1 (11.12)		77.2 (10.60)		71.0 (7.37)	
3M	79.7 (9.87) ^a^		-		-	
6M	80.0 (9.68) ^b^		75.9 (9.65) ^b^		-	
9M	80.3 (9.88) ^c^		-		-	
12M	80.2 (9.61) ^d^		76.1 (9.47) ^d^		71.8 (8.10) ^d^	
HDL-C, mg/dL		<0.001		0.002		<0.001
0M	41.2 (11.96)		51.5 (14.48)		62.8 (12.51)	
3M	42.4 (11.39) ^a^		-		-	
6M	43.8 (11.83) ^b^		51.0 (13.87) ^b^		-	
9M	44.8 (12.10) ^c^		-		-	
12M	45.2 (12.12) ^d^		52.6 (14.07) ^d^		60.5 (14.52) ^d^	
Triglycerides, mg/dL		<0.001		0.495		<0.001
0M	208.4 (116.04)		122.9 (70.05)		84.6 (26.38)	
3M	174.4 (98.77) ^a^		-			
6M	172.8 (95.06) ^b^		123.8 (68.13)			
9M	177.4 (102.43) ^c^		-			
12M	172.4 (96.62) ^d^		122.6 (66.06)		95.7 (46.34) ^d^	
Glucose, mg/dL		<0.001		0.016		<0.001
0M	106.1 (24.00)		95.7 (14.75)		88.9 (6.80)	
3M	101.1 (17.77) ^a^		-		-	
6M	100.2 (16.75) ^b^		94.0 (11.54) ^b^		-	
9M	101.3 (18.84) ^c^		-		-	
12M	101.2 (16.51)^d^		94.9 (11.72) ^d^		90.5 (9.15) ^d^	

SBP: Systolic blood pressure; DBP: Diastolic blood pressure; SD: Standard deviation; SMESY: Seoul Metabolic Syndrome Management; * *p* for linear trend using the linear mixed model after adjusting for age, gender, education, income, marital status, current smoking, physical activity, alcohol drinking, body mass index at baseline, and medications over 12 months; ^a^ *p* < 0.05: Significant difference between baseline and 3 months; ^b^ *p* < 0.05: Significant difference between baseline and 6 months; ^c^ *p* < 0.05: Significant difference between baseline and 9 months; ^d^ *p* < 0.05: significant difference between baseline and 12 months.

**Table 3:** Changes in the risk scores of metabolic syndrome over the SMESY intervention (*N* = 25,449).

Time (Month)	High-Risk Group (*n* = 7116)		Moderate-Risk Group (*n* = 14,762)		Low-Risk Group (*n* = 3571)	
	Mean (SD)	*p* *	Mean (SD)	*p* *	Mean (SD)	*p* *
Risk Score ^+^		<0.001		<0.001		<0.001
0M	0.63 (2.33)		−1.79 (0.28)		−6.72 (2.40)	
3M	−0.40 (2.39) ^a^		-		-	
6M	−0.34 (2.43) ^b^		−2.37 (2.36) ^b^		-	
9M	−0.14 (2.35) ^c^		-		-	
12M	−0.19 (2.30) ^d^		−2.15 (2.29) ^d^		−4.58 (2.37) ^d^	

SD: Standard deviation; SMESY: Seoul Metabolic Syndrome Management; ^+^ Risk score indicates sum of standardized scores of mean arterial pressure, high-density lipoprotein cholesterol, triglycerides, waist circumference and fasting glucose created from subtracting the individual risk factor from the NHLBI/AHA criteria and dividing by the sample standard deviation; * *p* for linear trend using the linear mixed model after adjusting for age, gender, education, income, marital status, current smoking, physical activity, alcohol drinking, body mass index at baseline, and medications over 12 months; ^a^ *p* < 0.05: Significant difference between baseline and 3 months; ^b^ *p* < 0.05: significant difference between baseline and 6 months; ^c^ *p* < 0.05: Significant difference between baseline and 9 months; ^d^ *p* < 0.05: Significant difference between baseline and 12 months.

**Table 4:** Changes in behavioral lifestyle factors over the SMESY intervention (*N* = 25,449).

Time (Month)	High-Risk Group (*n* = 7116)		Moderate-Risk Group (*n* = 14,762)		Low-Risk Group (*n* = 3571)	
	n (%) or Mean (SD)	*p* *	n (%) or Mean (SD)	*p* *	n (%) or Mean (SD)	*p* *
Current smokers		<0.001		<0.001		0.506
0M	1226/7072 (17.2)		1466/14,577 (10.1)		180/3531 (5.1)	
3M	642/4680 (13.7) ^a^		-		-	
6M	591/4529 (13.0) ^b^		944/11,949 (7.9) ^b^		-	
9M	281/2437 (11.5) ^c^		-		-	
12M	380/3279 (11.6) ^d^		704/8816 (8.0) ^d^		171/3407 (5.0)	
Physical activity		<0.001		<0.001		0.001
0M	1232/7097 (17.4)		2995/14,682 (20.4)		752/3550 (21.2)	
3M	972/4109 (23.7) ^a^		-		-	
6M	898/3972 (22.6) ^b^		2425/10,541 (23.0) ^b^		-	
9M	477/2140 (22.3) ^c^		-		-	
12M	633/2911 (21.7) ^d^		1837/7746 (23.7) ^d^		699/2967 (23.6) ^d^	
Healthy diet score ^+^		<0.001		<0.001		<0.001
0M	6.42 (2.36)		6.85 (2.30)		7.00 (2.32)	
3M	7.29 (2.21) ^a^		-		-	
6M	7.49 (2.18) ^b^		7.53 (2.21) ^b^		-	
9M	7.37 (2.10) ^c^		-		-	
12M	7.58 (2.17) ^d^		7.57 (2.15) ^d^		7.41 (2.22) ^d^	
Body weight, kg ^+^		<0.001		<0.001		0.011
0M	70.28 (12.26)		61.64 (10.19)		56.19 (7.96)	
3M	68.82 (11.99) ^a^		-		-	
6M	68.44 (11.80) ^b^		61.00 (9.93) ^b^			
9M	68.02 (11.77) ^c^		-		-	
12M	68.08 (11.51) ^d^		60.82 (9.86) ^d^		56.28 (8.09) ^d^	

SD: Standard deviation; SMESY: Seoul Metabolic Syndrome Management; Physical activity: moderate intensity activity ≥5 days per week; Alcohol drinking: Drinking ≥2 times per week; ^+^ Mean (SD); * *p* for linear trend using the linear mixed model after adjusting for age, gender, education, income, marital status, current smoking, physical activity, alcohol drinking, body mass index at baseline, and medications over 12 months; ^a^ *p* < 0.05: Significant difference between baseline and 3 months; ^b^ *p* < 0.05: Significant difference between baseline and 6 months; ^c^ *p* < 0.05: Significant difference between baseline and 9 months; ^d^ *p* < 0.05: Significant difference between baseline and 12 months.

**Table 5:** Changes in prevalence of metabolic syndrome over the SMESY intervention (*N* = 25,449).

Time (Month)	Prevalence		*β* (SE)	OR	95% CI	*p* *
	n	%				
0M	7116/25,449	28.0	-	1.00	-	-
12M	2722/15,948	10.7	–0.673 (0.035)	0.51	0.476–0.547	<0.001

*β* = unstandardized coefficient; CI: Confidence interval; OR: Odds ratio; SE: Standard error; SMESY: Seoul Metabolic Syndrome Management; p * obtained from generalized estimating equation (GEE) after adjusting for age, gender, education, income, marital status, current smoking, physical activity, alcohol drinking, body mass index, and medications over 12 months.
